# Prevalence and Reasons for Initiating Use of Electronic Cigarettes Among Adults in Montana, 2013

**DOI:** 10.5888/pcd11.140283

**Published:** 2014-11-20

**Authors:** Lisa Schmidt, Alison Reidmohr, Todd S. Harwell, Steven D. Helgerson

**Affiliations:** Author Affiliations: Alison Reidmohr, Todd S. Harwell, Steven D. Helgerson, Montana Department of Public Health and Human Services, Helena, Montana.

## Abstract

We used data from the 2013 Montana Adult Tobacco Survey to estimate the prevalence of electronic cigarette (e-cigarette) use and reasons for initiation among Montana adults. More than 1 in 10 (11.2%, 95% confidence interval [CI], 9.1%–13.2%) adults reported ever using e-cigarettes, and 1.3% (95% CI, 0.7%–1.9%) reported current use. Most respondents reported “trying something new” (64%) or “trying to quit or reduce cigarette use” (56%) as a reason for initiating use. Ongoing surveillance of these addictive products is needed.

## Objective

Cigarette smoking remains the leading cause of preventable death in the United States (1). Because of public health efforts, cigarette sales have steadily declined in Montana during past decades (2). Although tobacco use prevention programs continue to address cigarette use, other tobacco products are a growing concern. The tobacco industry is now marketing another tobacco product, the electronic cigarette (e-cigarette). Use of e-cigarettes quadrupled among US adults from 2009 to 2010 (3); however, little is known about why people are using this product. The objective of this study was to describe the prevalence of e-cigarette use and to identify reasons for initiating use among Montana adults.

## Methods

From January through June 2013 the Montana Department of Public Health and Human Services (DPHHS) conducted the Adult Tobacco Survey (ATS). The ATS is a population-based telephone survey of noninstitutionalized Montana adults conducted in collaboration with the Centers for Disease Control and Prevention (CDC). The sample was selected by random-digit dialing from lists of cellular and landline telephone numbers.

Participants were selected anonymously, and more than 5,000 Montanans participated in the survey. The participation rate (included only answered telephone calls) was 57.8% (3,311 of 5,729) for the landline sample and 66.9% (1,756 of 2,625) for the cellular sample. These rates are consistent with the participation rates of Adult Tobacco Surveys conducted in other states (4,5). The ATS collects detailed information about tobacco use and beliefs and attitudes about its use. Most questions in the survey were validated questions provided by CDC’s Office on Smoking and Health.

The survey included 3 questions about e-cigarette use. The first 2 questions were, “Have you ever used an electronic cigarette, even just one time in your entire life?” and “Do you now use electronic cigarettes every day, some days, rarely, or not at all?” We classified respondents who reported ever using e-cigarettes and who reported using e-cigarettes every day or some days as current e-cigarette users. The third question was for any e-cigarette user and asked respondents to select all of the reasons they initiated use of e-cigarettes. The response categories were to quit smoking cigarettes, to reduce cigarette consumption, to try something new (curiosity), to not disturb other people with smoke, to smoke in a place where cigarette smoking is banned, to save money, e-cigarettes might be less harmful than cigarettes, e-cigarettes taste better, and other.

Data were analyzed using SAS statistical software, version 9.3 (SAS Institute, Inc). Weighted prevalence estimates and 95% confidence intervals (CIs) were calculated. Chi-square tests were used to compare the prevalence rates and reasons for initiating use by selected demographic characteristics.

## Results

The Council of American Survey Research Organizations (CASRO) response rates, which included all attempted telephone numbers, were 47% for landline telephone respondents and 25% for cellular telephone respondents. Overall, less than 2% of adult respondents reported current use of e-cigarettes (1.3%; 95% CI, 0.7%–1.9%) (data not shown). However, 11.2% (95% CI, 9.1%–13.2%) of Montana adults reported they had ever used e-cigarettes, and the prevalence varied significantly by age. The median age of adults who reported use of e-cigarettes was 30 years (95% CI, 27–34 y). Among respondents who reported ever use of e-cigarettes, 71% were current cigarette smokers (data not shown). Ever use of e-cigarettes was higher among younger adults than older adults (Table). Among respondents who were current cigarette smokers, over half reported ever using e-cigarettes, markedly higher than the prevalence of e-cigarette use reported by former and noncigarette users. Almost 10% of cigarette smokers also reported current use of electronic cigarettes (data not shown). There was no significant difference in the prevalence of e-cigarette use by sex or by race (Table).

**Table:** Electronic Cigarette (e-Cigarette) Use Among Adults (N = 5,135), Montana Adult Tobacco Survey, 2013

Characteristic	% (95% Confidence Interval)
**Ever e-cigarette use**	11.2 (9.1–13.2)
**Age, y**	
18–34	22.5 (16.4–28.6)
35–54	9.8 (6.8–12.8)
≥55	4.1 (2.8–5.4)
**Sex**	
Male	11.7 (8.4–15.0)
Female	10.7 (8.1–13.2)
**Race**	
White	10.5 (8.3–12.6)
American Indian/Alaska Native	18.8 (11.6–26.0)
**Cigarette smoking status**	
Current	55.6 (45.8–65.4)
Former	8.2 (5.4–11.1)
Never	1.7 (0.9–2.5)

**Figure 1:**
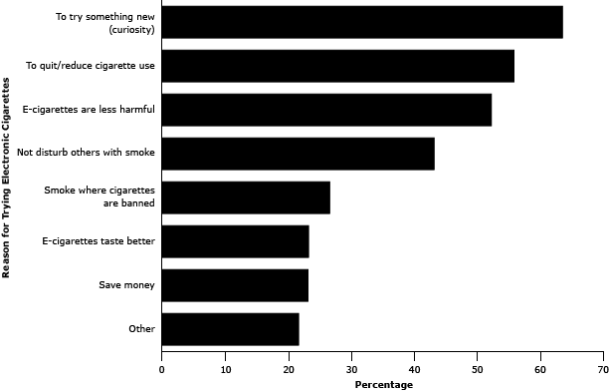
Reasons for trying electronic cigarettes among Montana adults, Montana Adult Tobacco Survey, 2013. **Table:** 

**Figure 2:**
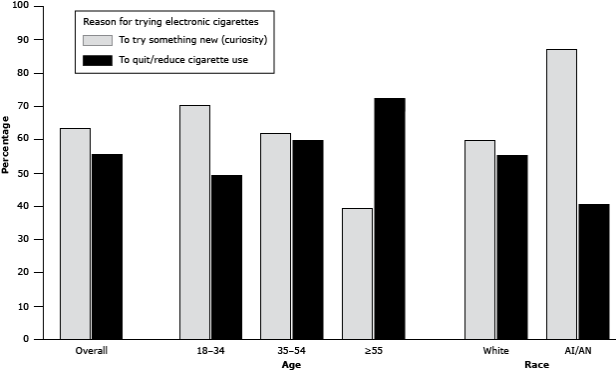
Reasons for trying electronic cigarettes among Montana adults, by age and race. Montana Adult Tobacco Survey, 2013. Abbreviation: AI/AN, American Indian/Alaska Native. **Table:** 

The most frequently reported reasons for initiating e-cigarette use were “to try something new (curiosity)” (64%) and “to quit/reduce cigarette use” (56%) (Figure 1). Just over half of respondents indicated initiating use because “e-cigarettes are less harmful than cigarettes” (52%). Fewer respondents tried e-cigarettes because of smoke restrictions, taste preference, or cost savings. Most younger adult e-cigarette users reported they wanted to try something new (Figure 2). In contrast, older adult e-cigarette users were more likely to report they wanted to quit or reduce cigarette use. Curiosity was also the most frequent reason reported for trying e-cigarettes by both white and American Indian/Alaska Native e-cigarette users.

Reason for Trying Electronic Cigarettes	Percentage
Try something new (curiosity)	64
Quit/reduce cigarette use	56
E-cigarettes are less harmful	52
Not disturb others with smoke	43
Smoke where cigarettes are banned	26
E-cigarettes taste better	23
Save money	23
Other	22

Characteristic	To Try Something New (Curiosity), %	To Quit/Reduce Cigarette Use, %
Overall	64	56
**Age, y**		
18–34	70	50
35–54	62	60
≥55	39	73
**Race**		
White	60	55
American Indian/Alaska Native	87	41

## Discussion

Our results are consistent with findings from other states. In 2012, the prevalence of current e-cigarette use among adults was 1.8% (95% CI, 1.4%–2.2%) in California and 1.9% (95% CI, 1.3%–2.8%) in Alaska (6). Although the prevalence of current e-cigarette use is low, more than 20% of young adults in Montana have tried e-cigarettes. Twenty-four percent of this population also reported current cigarette use, indicating the need for effective prevention programs for all nicotine products, specifically those that are marketed to young adults.

Most younger e-cigarette users reported they initiated use of e-cigarettes because they wanted to try something new. By contrast, older e-cigarette users wanted to quit or reduce cigarette use. This finding is exceptional, because previous studies have shown quitting smoking or reducing harm to be the leading reasons for using e-cigarettes (7,8). If most young adults are using e-cigarettes without intention to quit smoking, e-cigarettes may create new or dual users.

This study has several limitations. First, the ATS is a telephone survey of noninstitutionalized adults; therefore, people without telephones and those who are institutionalized are not represented in the sample. Also, because ATS data are self-reported, they are subject to recall and social desirability bias.

Considering that e-cigarettes have only recently entered the market, our findings show that ever use of e-cigarettes by adults is prevalent, and reasons for initiating use differ by age group. Experimentation with e-cigarettes could lead to use of other tobacco products; it is important to monitor for dual use, as well as initiation of nicotine addiction (9–11) Given the lack of evidence indicating what the short- and long-term health effects of e-cigarettes may be, further research is needed on adult and youth use patterns. State tobacco control programs should conduct ongoing surveillance of e-cigarettes to monitor use and to inform health education strategies.
